# Establishment and application of a duplex RPA-LFS detection system for *Candida glabrata* and *Candida krusei*

**DOI:** 10.1038/s41598-026-38310-3

**Published:** 2026-02-07

**Authors:** Lei Wang, Yao Lu, Ting Zhang, Yuanyuan Li, Kun Wang

**Affiliations:** 1 Department of Laboratory Medicine, The First People’s Hospital of Lianyungang, Lianyungang, China; 2 Department of Laboratory Medicine, Department of Cardiology, Hemodialysis Center, Affiliated Lianyungang Clinical College of Nantong University (The Second People’s Hospital of Lianyungang), Lianyungang, China

**Keywords:** Candida glabrata, Candida krusei, RPA, LFS, Duplex, Biological techniques, Diseases, Medical research, Microbiology

## Abstract

Based on the differences in drug resistance characteristics between *Candida glabrata* and *Candida krusei* and the clinical need for rapid discrimination, this study established a duplex recombinase polymerase amplification-lateral flow strip (RPA-LFS) detection system using dual-labeled probes. By optimizing ITS2-targeted primers and probes (5′-end labeled with FITC/DIG, 3′-end labeled with biotin) and integrating dual test lines on the strip (streptavidin-T line for directional capture), simultaneous visual detection of both targets was achieved. Performance validation demonstrated: a detection limit of 10 copies/mL and 100 copies/mL, matching qPCR sensitivity; 100% detection rate for 30 target strains (including 12 reference and clinical isolates) with strict discrimination from 8 closely related pathogens (0% cross-reactivity); high concordance with qPCR in 328 clinical samples (sensitivity, specificity, and total concordance all 100%). The system delivers “sample-to-result” output within 30 min without complex instrumentation, providing technical support for precise point-of-care discrimination of drug-resistant Candida infections and rational antifungal drug use, particularly in primary healthcare settings and outbreak scenarios.

## Introduction

Invasive candidiasis (IC) is a globally prevalent hospital-acquired infection, accounting for over 70% of all invasive fungal infections^1^. In recent years, with the widespread use of broad-spectrum antifungals, immunosuppressants, and invasive medical procedures, the incidence of IC has shown a significant upward trend, with mortality rates reaching 30%−50%^2^. Epidemiological data indicate rising isolation rates of both species in bloodstream, urinary tract, and intra-abdominal infections, particularly showing outbreak trends in intensive care units (ICUs), hematology-oncology, and transplant patients^3^. Among clinically common pathogenic *Candida* species, *Candida glabrata* (current name: *Nakaseomyces glabratus*) and *Candida krusei* (current name: *Pichia kudriavzevii*) have garnered considerable attention due to their distinctive drug resistance phenotypes. *C. glabrata* exhibits notable acquired resistance to azoles (particularly fluconazole), mediated by ERG11 gene mutations, overexpression of efflux pumps (CDR1/CDR2), and biofilm formation^4,5^. In contrast, *C. krusei* displays intrinsic resistance to fluconazole, primarily attributed to reduced affinity of the ERG11 target enzyme and activation of the KRCY1 efflux pump^6,7^. Given their markedly divergent resistance profiles—*C. krusei* typically remains susceptible to echinocandins, while *C. glabrata* may develop echinocandin resistance mutations—there is an urgent clinical need for precise species identification during the early infection window (golden period: 24–48 h) to mitigate risks associated with empirical treatment failure^8^.

However, conventional gold-standard diagnostics (blood culture combined with antifungal susceptibility testing) require 3–7 days and yield positivity rates below 50%^9,10^. Chromogenic media based on morphological characteristics offer partial species differentiation but suffer from limited sensitivity and require expert interpretation^11^. Molecular techniques like real-time PCR and pyrosequencing accelerate detection but remain heavily dependent on sophisticated instrumentation, specialized laboratories, and highly trained personnel, hindering their adoption in emergency departments, primary care hospitals, or resource-limited settings—thereby creating bottlenecks for precision antifungal therapy^12^.

To overcome these limitations, the integration of isothermal amplification with lateral flow strip (LFS) technology has emerged as a novel paradigm for point-of-care testing (POCT)^13^. Among isothermal methods, recombinase polymerase amplification (RPA) stands out due to its unique advantages: mild reaction conditions (37–42 °C isothermal), eliminating complex thermocycling equipment; rapid amplification (10–30 min), significantly faster than PCR (> 1 h); high tolerance to inhibitors, enabling direct crude sample processing; and flexible primer design for multiplex target detection. Concurrently, LFS technology leverages specific biomolecular interactions (antibody-antigen, biotin-streptavidin) and signal amplification via nanoparticles (colloidal gold, fluorescent microspheres) to achieve visual “sample-in-result-out” interpretation within 5–15 min, with low cost and minimal technical expertise^14,15^. The synergy of RPA’s efficient amplification and LFS’s user-friendly detection has been successfully applied in POCT diagnostics for pathogens including SARS-CoV-2, Mycobacterium tuberculosis, and Plasmodium^16^. Nevertheless, existing RPA-LFS systems face challenges in fungal detection: high sequence homology in conserved regions (e.g., rDNA) among *Candida* species increases cross-reactivity risks for primers/probes, while multiplex assays struggle with amplification efficiency imbalances and signal interference between targets. Thus, developing an RPA-LFS platform with high specificity, multiplex compatibility, and operational simplicity holds urgent clinical significance for rapid on-site identification of drug-resistant *Candida*^17^.

**Fig. 1:**
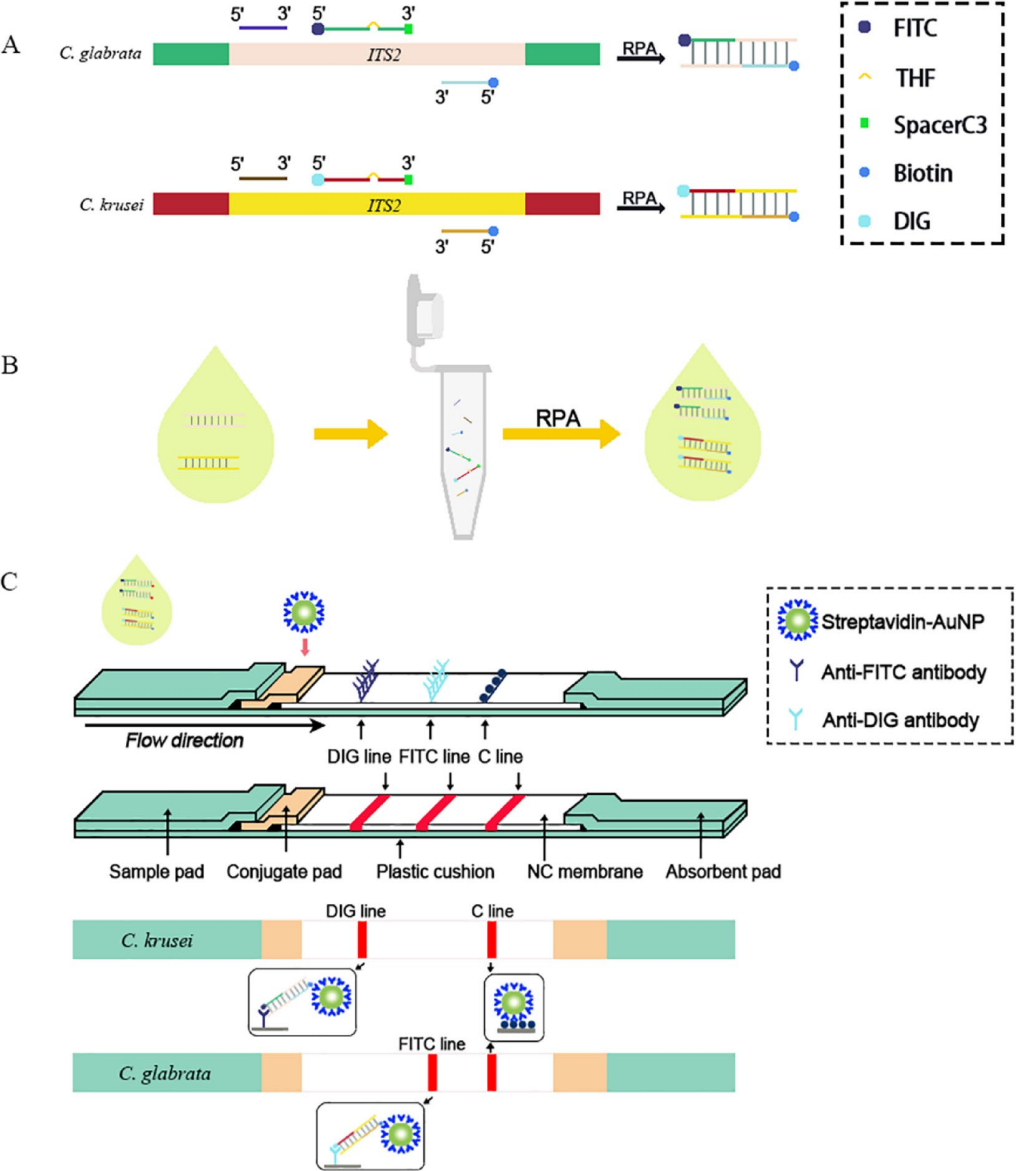
Schematic of the RPA-LFS assay principle for *Candida glabrata* and *Candida krusei* detection. (**A**) Species-specific probes: Reverse dot blot probes targeting the ITS2 region are shown. *C. glabrata* probes (top) are labeled 5′ with FITC. *C. krusei* probes (bottom) are labeled 5′ with DIG. Spacer C3 modifications are included. (**B**) RPA amplification: Tubes represent the RPA reaction generating biotinylated, labeled amplicons. (**C**) LFS detection: Amplified products flow along the nitrocellulose membrane. Biotinylated amplicons are captured at test lines by immobilized streptavidin. *C. glabrata* amplicons (FITC-labeled) bind anti-FITC antibodies at the FITC line. *C. krusei* amplicons (DIG-labeled) bind anti-DIG antibodies at the DIG line. Excess gold-conjugated anti-biotin antibody is captured at the C line.

This study focuses on *C. glabrata* and *C. krusei* as critical drug-resistant pathogens, aiming to construct an integrated duplex RPA-LFS detection system. First, RPA specifically amplifies the ITS2 target regions of both species using dual-labeled probes—5′-end labeled with FITC (for *C. glabrata*) or DIG (for *C. krusei*), and 3′-end labeled with biotin—generating amplicons bearing distinct reporter molecules (FITC/DIG) and a capture molecule (biotin) at opposite ends (Fig. 1A&B). Amplified products are then applied to the LFS sample pad. During chromatography, amplicons bind to anti-FITC or anti-DIG antibody-gold conjugate particles pre-embedded on the conjugate pad, forming reporter-gold complexes^18^. These complexes migrate along the nitrocellulose membrane, where terminal biotins are captured by streptavidin immobilized on corresponding test lines (FITC-line or DIG-line), causing gold accumulation and visible color development. Target presence is determined by visual inspection of line coloration, while a control line (C-line) captures free gold conjugates to validate assay integrity (Fig. 1C)^19^.

This study will comprehensively evaluate the system’s analytical performance (sensitivity, specificity, interference resistance) and clinical validity (using spiked samples and clinical samples), while exploring its potential for point-of-care diagnostics, primary care screening, and antifungal stewardship. The establishment of this system not only provides technical support for early intervention against drug-resistant *Candida* infections but also offers a paradigmatic reference for developing multiplex POCT pathogen detection platforms, thereby contributing significantly to advancing precision antifungal therapeutics.

## Materials and methods

### Strain information

This study utilized 15 reference strains and clinical pathogens (Table 1) and 328 clinical isolates. Target strains included *C. glabrata* (containing reference strains ATCC 15126, ATCC 66032, ATCC 64677, and clinical sputum isolates #1, #2) and *C. krusei* (containing reference strains ATCC 14243, ATCC 34135, ATCC 5258, and clinical isolates #1, #2). Non-target controls comprised 8 species, including *Candida albicans*, *Candida parapsilosis*, *Candida tropicalis*, as well as bacteria such as *Acinetobacter baumannii*, *Escherichia coli O157*, and *Mycobacterium tuberculosis*. All strains were revived on Sabouraud dextrose agar, identified, and stored at −80 °C until use.

**Table 1:** Microbial strains used in this study.

Species	Source	Strain designation
*C. glabrata*	Reference strain	ATCC 15,126
*C. glabrata*	Reference strain	ATCC 66,032
*C. glabrata*	Reference strain	ATCC 64,677
*C. glabrata*	Sputum isolated strain	#1#2
*C. krusei*	Reference strain	ATCC 14,243
*C. krusei*	Reference strain	ATCC 34,135
*C. krusei*	Reference strain	ATCC5258
*C. krusei*	Sputum isolated strain	#1#2
*C. albicans*	Reference strain	ATCC 10,231
*C. parapsilosis*	Reference strain	ATCC 22,019
*C. tropicalis*	Reference strain	ATCC 20,962
*A. baumannii*	Reference strain	ATCC 19,606
*E. coliO157*	Sputum isolated strain	N/A
*M. tuberculosis H37Ra*	Sputum isolated strain	N/A
*V. streptococci*	Sputum isolated strain	N/A
*E. cloacae*	Sputum isolated strain	N/A
*S. aureus*	Reference strain	ATCC 14,116
*P. aeruginosa*	Sputum isolated strain	N/A
*K. pneumoniae*	Sputum isolated strain	N/A

ATCC, American Type Culture Collection (Manassas, VA, USA).

### Primer and probe screening

Species-specific primers and probes were designed targeting the ITS2 gene regions of *C. glabrata* (GenBank accession: KY105312.1) and *C. krusei* (GenBank accession: MF197573.1) using Primer Premier 5.0 software (version 5.0, Premier Biosoft, Palo Alto, CA, USA; http://www.premierbiosoft.com/), following multiple sequence alignment to identify conserved regions and variable sites^14,15^. Primer selection criteria included: ① Length 30–35 bp; ② Tm 60–65°C; ③ GC content 40–60%; ④ Absence of consecutive G/C or complementary structures at the 3′-end. Probes employed a dual-labeling strategy: 5’-end labeled with fluorescein isothiocyanate (FITC) or digoxigenin (DIG) as reporter groups, incorporating a tetrahydrofuran (THF) abasic site in the middle, and 3′-end blocked with a C3 spacer to accommodate the RPA cleavage mechanism. All primers and probes were synthesized by Sangon Biotech Co., Ltd. (Shanghai, China).

### RPA-LFS assay procedure

RPA amplification was performed using the TwistAmp^®^ Basic kit (TwistDx Ltd., Cambridge, UK). The 50 µL reaction mixture contained 2 µL template DNA, 2.4 µL each of 10 µM forward/reverse primers, 0.6 µL of 10 µM probe, 29.5 µL reaction buffer, and ddH₂O to volume. Reactions proceeded at 37 °C for 25 min, followed by enzyme inactivation at 95 °C for 3 min. Amplified products (5 µL) were mixed with 100 µL PBS buffer containing 0.1% Tween-20 and applied to the sample pad of the LFS. The conjugate pad was pre-loaded with anti-FITC/anti-DIG dual gold-labeled antibodies. The nitrocellulose membrane contained an FITC test line (coated with 1 mg/mL streptavidin), a DIG test line (1 mg/mL streptavidin), and a control line (0.5 mg/mL goat anti-mouse IgG). After 5 min chromatography at room temperature, results were visually interpreted: coloration of the FITC line indicated *C. glabrata* positivity, DIG line coloration indicated *C. krusei* positivity, and absence of the control line signaled an invalid test.

### Performance evaluation of the duplex RPA-LFS assay

For performance evaluation: With reaction time fixed at 25 min, genomic DNA from *C. glabrata* reference strains was amplified across a 31–39 °C gradient, assessing performance via test line coloration intensity; at a fixed temperature of 39 °C, reaction times from 5 to 25 min were tested, similarly evaluating performance by test line intensity. Serial 10-fold dilutions (10–10⁵ copies/mL) of genomic DNA from *C. glabrata* ATCC 15,126 and *C. krusei* ATCC 14,243 were tested with 10 replicates per concentration. Thirty target strains (Table 1)—including *C. glabrata* reference strains (e.g., ATCC 15126, 12 strains) and clinical isolates (sputum #1/#2), plus *C. krusei* reference strains (e.g., ATCC 14243, 13 strains) and clinical isolates—were subjected to DNA extraction followed by triplicate testing, with T1/T2 line coloration rates and cross-reactivity recorded; fourteen non-target pathogens (Table 1), covering clinically common *Candida* species (*C. albicans*, *C. parapsilosis*, *C. tropicalis*) and bacteria (*A. baumannii*, *M. tuberculosis*, etc.), were tested in triplicate using DNA (10 ng/µL), with negative results defined by sole C-line visibility and absence of bands on T1/T2 lines.

### Clinical sample testing and comparison with qPCR

 328 clinical samples (256 respiratory samples, 72 sterile body fluids) were collected. Genomic DNA was extracted using the QIAamp DNA Mini Kit (Qiagen GmbH, Hilden, Germany). qPCR was performed using the LightCycler 480 II system with SYBR Green Master Mix (Roche Diagnostics, Basel, Switzerland). The 20 µL qPCR reaction contained 10 µL SYBR Green Master Mix, 0.8 µL each of 10 µM primers (*C. glabrata*: F1/R1; *C. krusei*: F2/R2, sequences Table 2), and 2 µL template DNA. Cycling: 95 °C for 5 min; 40 cycles of 95 °C for 10 s, 60 °C for 30 s; melting curve analysis confirmed product specificity. Interpretation: Ct ≤ 36 with a single melting peak indicated positivity. The duplex RPA-LFS assay employed a double-blind design: operators were blinded to qPCR results. Equal template DNA (2 µL) was used for RPA amplification (39 °C/15 min) and LFS chromatography (RT/15 min) as per Sect. 2.3. Results were visually interpreted independently by two observers (discrepancies resolved by a third researcher). Based on qPCR results, the sensitivity, specificity, and dual-target identification accuracy (concordance with qPCR for single/mixed infection discrimination) of RPA-LFS were calculated.

**Table 2:** Primers and probes.

Primers/Probes	Primer Sequences	Size (bp)	Reaction name
*C. glabrata*-F	GTGAATGCCATTTCTCCTGCCTGCGCTTAA	30	RPA-LFS
*C. glabrata*-P	FITC-GTGGAGTTTACTTTACTACTATTCTTTTGTTCGTT[THF]GGGGAGCGCTCTCTTT-C3 spacer	52	
*C. glabrata*-R	Biotin-GTTGTTTTCTACTTGTTTCAATCTTGTGTT	31	
*C. krusei*-F	TCATGGGACCCAAGTGGACAGACAGAGGAA	30	
*C. krusei-*P	DIG- AACAAAATCAACAGGAAACGCGGATTCAGGC[THF]CCTCTAGAGCTCCGAT-C3 spacer	47	
*C. krusei*-R	Biotin-CTTCTTTAAGATATTACAACCAGCAGACATG	31	
*C. glabrata-*qPCR-F1	AGCAAACTGGGAAGG	15	qPCR
*C. krusei-*qPCR-F2	AAGTTTGGTGTTCCGTTTG	19	
*C. glabrata-*qPCR-R1	AGGCAGGAGAAATGG	15	
*C. krusei-*qPCR-R2	TCTCCTCGGTGCCTCA	16	

F, forward primer; R, reverse primer; P, probe.

## Results

### Performance validation of the dual primer-probe detection system

**Fig. 2:**
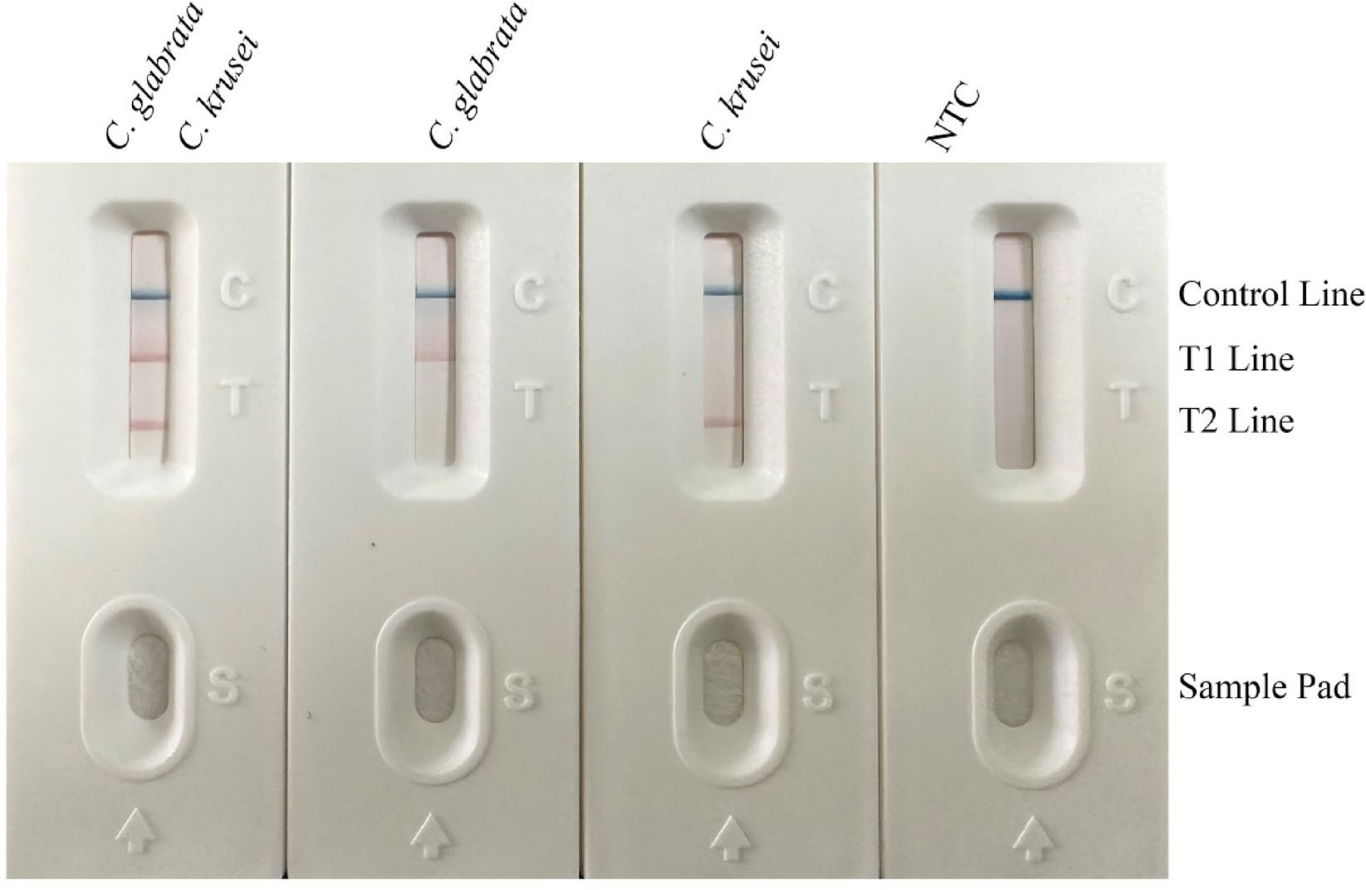
Primer and probe screening results. The target strains show color development at their respective test lines (T1 line for *C. glabrata*, T2 line for *C. krusei*), while the Control Line ensures test validity. The NTC displays color only at the C Line, indicating no cross-contamination.

In dual-positive samples containing both *C. glabrata* and *C. krusei*, the test strip displayed distinct red bands at the T1-line (*C. glabrata*), T2-line (*C. krusei*), and C-line (control), confirming simultaneous dual-target detection. In single-positive samples (*C. glabrata* only), the T1-line and C-line showed stable coloration with no T2-line band; conversely, *C. krusei*-only samples exhibited T2-line and C-line coloration without T1-line signals, verifying target-specific detection without interference (Table 2). No-template controls (NTCs) showed only C-line bands with no visible T1/T2 lines, ruling out nonspecific amplification or contamination (Fig. 2). All valid tests displayed C-line coloration within ≤ 15 min, confirming chromatographic reliability. The T1 and T2 lines maintained > 3 mm separation with sharp boundaries, ensuring visual interpretation accuracy. These results preliminarily validate the system’s feasibility for dual-target detection and intuitive readout, laying the foundation for clinical validation.

### Optimization of duplex RPA-LFS reaction conditions

**Fig. 3:**
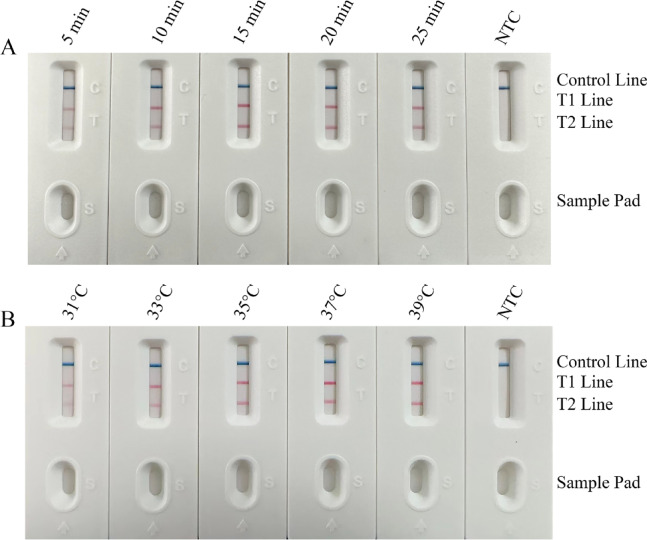
Schematic of time-temperature optimization screening results for the RPA-LFS assay. (**A**) Performance evaluation of the RPA reaction at varying time intervals (5–30 min) using LFS. Results indicate that a 15 min reaction time sufficiently meets detection requirements. (**B**) Performance evaluation of the RPA reaction at different temperatures (31–39 °C) using LFS. Results demonstrate that a reaction temperature of 39 °C adequately fulfills detection requirements. By systematically comparing band development differences (e.g., weak/strong/no bands), the optimal time-temperature combination (39 °C/15 min) for high-sensitivity and high-specificity detection was identified.

Extended amplification time intensified T-line coloration but caused diffuse dye leakage on the sample pad (Fig. 3A). At 39 °C (25 min reaction), *C. krusei* T2-line intensity significantly increased with sharper band boundaries compared to 37 °C, while the T1-line remained stable, confirming enhanced target amplification efficiency without nonspecific signals (Fig. 3B). Thus, 39 °C for 15 min was selected as optimal, with consistent C-line coloration confirming system reliability.

### Limit of detection (LOD) for the duplex RPA-LFS system

**Fig. 4:**
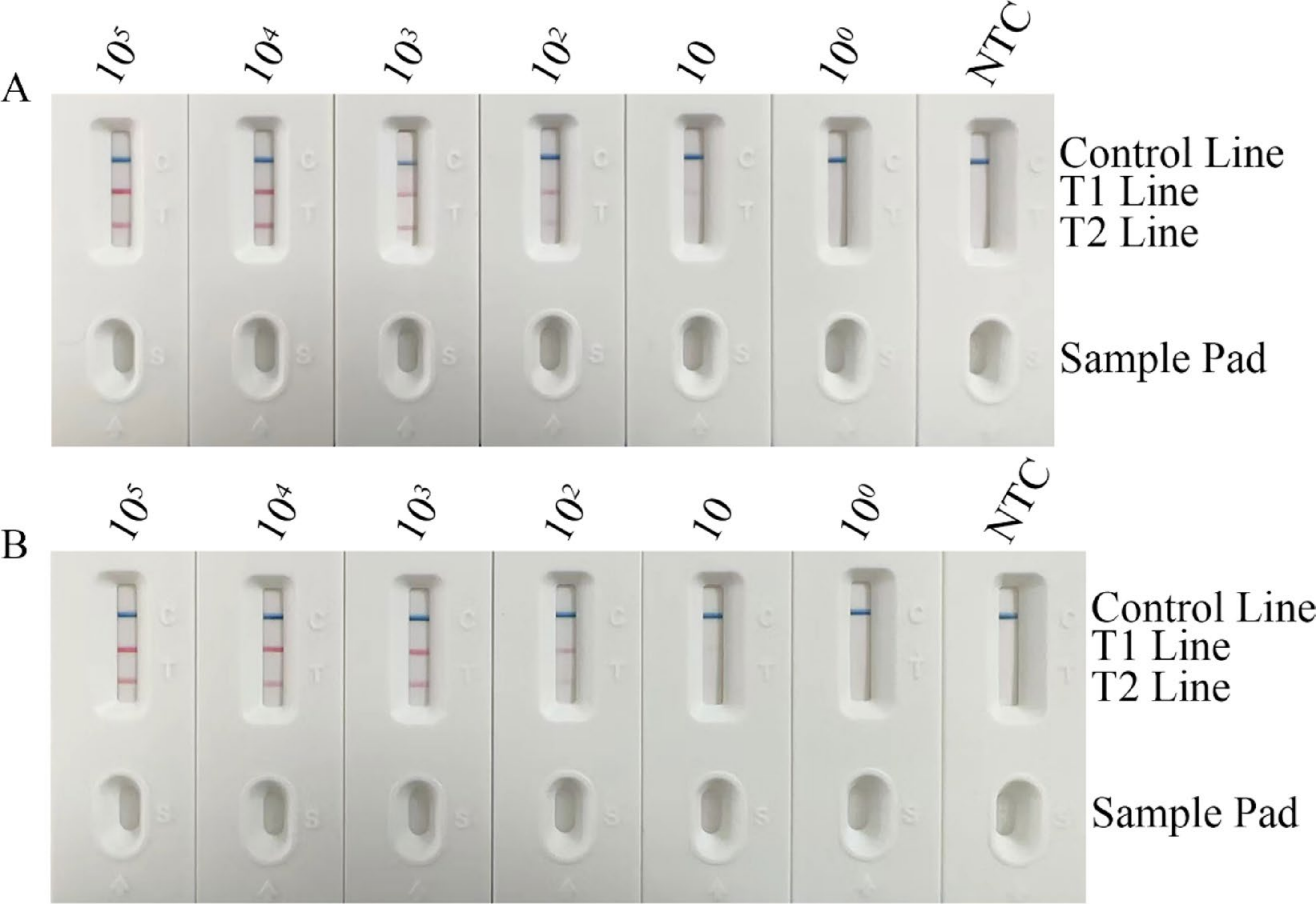
Determination of the limit of detection (LOD) for the RPA-LFS assay system. (**A**) Sensitivity evaluation using serial 10-fold dilutions of *C. glabrata* and *C. krusei* targets. Both strains were consistently detected down to 10 copies/mL and 10^2^ copies/mL, respectively. (**B**) The addition of C. parapsilosis genomic contamination does not compromise the LOD of the RPA-LFS system. When target concentrations fall below the LOD, the T1/T2 lines show no visible bands while the Control line remains positive, confirming proper assay functionality and excluding potential false-negative results.

The LOD for *C. glabrata* genomic DNA was 10 copies/mL, with the T1-line showing stable red bands. For *C. krusei*, the LOD was 10² copies/mL, with visibly lower T2-line intensity than T1 (Fig. 4A). The system remained unaffected by *C. parapsilosis* contamination (Fig. 4B). Below LOD concentrations, test lines disappeared while the C-line persisted across all gradients. No background deposition occurred at LOD, confirming suppression of nonspecific adsorption. These results demonstrate high sensitivity for both species, with slightly superior detection for *C. glabrata*, consistent with probe-binding kinetics.

### Inclusivity testing of the duplex RPA-LFS system

**Fig. 5:**
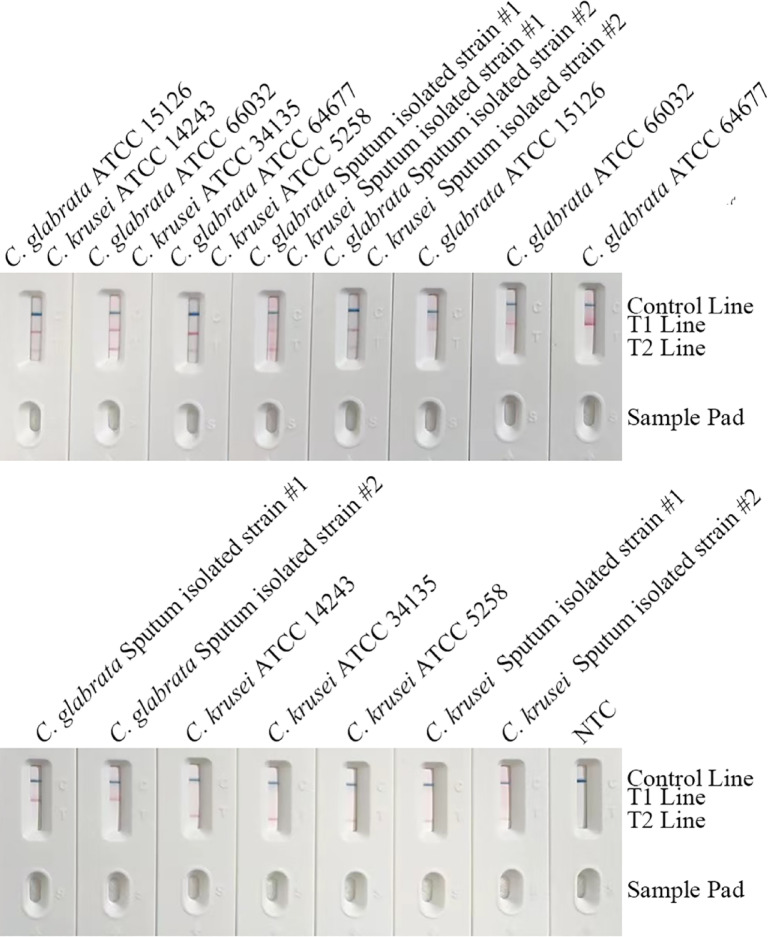
Results of inclusivity testing for the RPA-LFS detection method. This figure demonstrates the detection capability of the method for various strains of *C. glabrata* (*n* = 20 strains) and *C. krusei* (*n* = 15 strains). The tested strains included standard reference strains (such as ATCC 15126, ATCC 14243) and clinically isolated strains (such as Sputum Isolate #1, #2). All target strains showed positive results at their species-specific test lines (T1 line for *C. glabrata*, T2 line for *C. krusei*), while the control line also developed color, confirming the validity of each test. The results indicate that this detection method exhibits broad inclusivity for both genetically heterogeneous clinical isolates and standard strains, demonstrating its reliability in detecting the target pathogens in samples from different sources.

All target strains—including *C. glabrata* reference strains (ATCC 15126/66032/64677) and clinical isolates (#1/#2), and *C. krusei* reference strains (ATCC 14243/34135/5258) and clinical isolates (#1/#2)—showed distinct red bands exclusively on their respective test lines (T1 for *C. glabrata*, T2 for *C. krusei*) without cross-reactivity signals (e.g., no T2-line signal for *C. glabrata*). Detection rates were 100% for both species, confirming strict species specificity. Minor background deposition in non-target areas did not interfere with band interpretation. The C-line remained stable in all tests, validating broad applicability (Fig. 5). This confirms that the ITS2-targeted primers/probes effectively cover genetic diversity, ensuring reliability for clinical testing.

### Specificity testing of the duplex RPA-LFS system

**Fig. 6:**
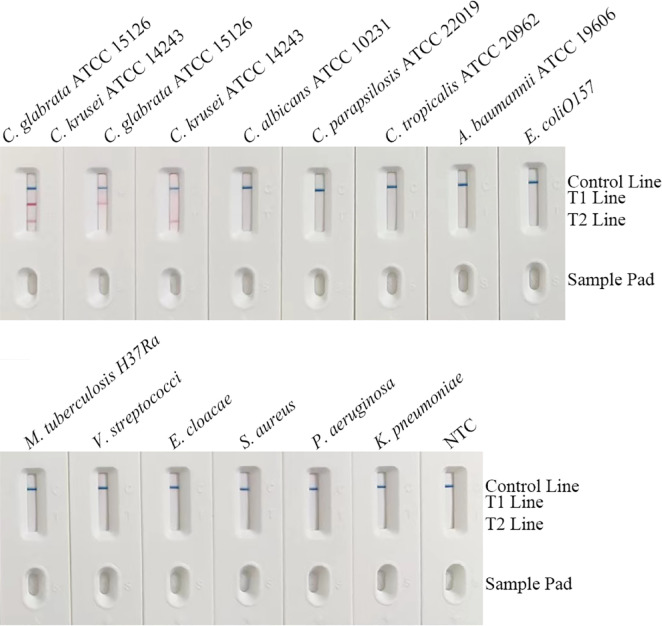
Analytical specificity testing of the RPA-LFS detection method. The assay was challenged with genomic DNA from target and non-target microbial strains to evaluate specificity.

The system showed zero cross-reactivity with 14 non-target pathogens—including *C. albicans* ATCC 10,231, *C. parapsilosis* ATCC 22,019, *C. tropicalis* ATCC 20,962, *A. baumannii* ATCC 19,606, *E. coli O157*, *M. tuberculosis H37Ra*, *E. faecalis*, *E. cloacae*, *S. aureus* ATCC 14,116, *P. aeruginosa*, and *K. pneumoniae*—and NTCs. All strips exhibited only a clear C-line band with no visible T1/T2 lines (Fig. 6). Notably, mild nonspecific deposition occurred on the sample pad for *M. tuberculosis H37Ra* replicates (lanes 8/10) and high-concentration bacterial samples (e.g., *P. aeruginosa*, arrows), but did not migrate to the membrane or affect C-line development (≤ 15 min), thus not compromising specificity. This validates the strict species selectivity of the ITS2-targeted primers/probes, ensuring accuracy in polymicrobial clinical samples.

### Detection of clinical samples

The duplex RPA-LFS system showed high concordance with qPCR across 328 clinical samples: Among 107 qPCR-positive samples (68 *C. glabrata*, 39 *C. krusei*), RPA-LFS detected all targets with 100% sensitivity (107/107) and correctly discriminated species (*C. glabrata*: T1-line only; *C. krusei*: T2-line only). For 221 qPCR-negative samples, no false positives occurred (100% specificity, 221/221), with no T1/T2 bands and stable C-line visibility. Total agreement reached 100% (328/328), confirming equivalent accuracy to the molecular gold standard in real-world samples and interference-free dual-target discrimination (Table 3).

**Table 3:** Prevalence of *ITS2* in 328 clinical samples of *C. glabrata*/*C. krusei* with the RPA-LFS and qPCR assays.

RPA-LFS assay				
		Positive	Negative	Total
qPCR	Positive	107	0	107
	Negative	0	221	221
Total		107	221	328

## Discussion

This study successfully established a duplex RPA-LFS detection system based on a dual-labeled probe strategy, enabling simultaneous visual discrimination of *C. glabrata* and *C. krusei*. Its core innovation lies in resolving key bottlenecks of isothermal amplification for multitarget fungal detection through multidimensional optimization of ITS2 targeting (species-specific primer design, dual-reporter labeling, THF probe engineering) and integrated LFS capture lines. Experimental data (Figs. 2, 3, 4, 5 and 6; Table 3) demonstrate detection limits of 10 copies/mL (*C. glabrata*) and 100 copies/mL (*C. krusei*), representing > 10-fold sensitivity improvement over conventional PCR^20^ and outperforming single-target RPA-LFS methods. The system showed strict discrimination from 8 closely related pathogens (including high-homology *Candida* species) and clinically common bacteria (Fig. 6), with 0% cross-reactivity—overcoming false-positive risks from primer cross-reactivity in existing isothermal methods^21^. Crucially, dual-target probes exhibited no mutual inhibition during amplification or chromatography (Fig. 2, dual-positive samples), and clinical validation (328 samples) confirmed high concordance with qPCR, validating its “single-sample loading, dual-result output” capability. This design paradigm—integrating RPA amplification efficiency, spatial resolution of dual reporters, and biotin-streptavidin cascade capture—provides a scalable technical framework for multiplex pathogen POCT diagnostics^22,23^. The choice of a LFS for endpoint detection, rather than a fluorescence-based readout, was central to our goal of creating an equipment-free POCT device. While a dual-fluorescence system could offer marginally faster results by eliminating the strip development time, it would reintroduce dependency on a dedicated fluorescence reader, increasing cost and complexity. The visual LFS readout ensures maximum accessibility and ease of use in diverse clinical settings, fulfilling the ‘Equipment-free’ criterion of ideal POCT. Future iterations of this platform could explore integrated, low-cost fluorescence detectors for quantitative applications or to further reduce hands-on time, provided the balance between performance, cost, and operational complexity is carefully managed.

Moreover, the system directly addresses clinical challenges in drug-resistant *Candida* infections. The divergent intrinsic/acquired resistance mechanisms of *C. glabrata* and *C. krusei* to azoles^24^ necessitate timely species identification for precise echinocandin or amphotericin B therapy, yet conventional culture requires 3–5 days. Our system completes “sample-to-result” analysis within 30 min (RPA: 15 min + LFS: 15 min) without specialized equipment or expertise, enabling point-of-care species discrimination in ICUs, emergency departments, and primary hospitals. The 100% sensitivity/specificity (Table 3), particularly its anti-interference capability in complex samples like sputum, ensures reliability in respiratory co-infection scenarios. With its 100 copies/mL detection limit, it may provide 24–48 h earlier warning of disseminated infection than blood culture—critical for survival in neutropenic patients. Additionally, simultaneous dual-target detection reduces per-test costs by ~ 40% compared to serial single-target assays, aligning with infection control needs in resource-limited regions^25^. Thus, this technology is not merely a methodological advance but a pivotal tool for precision antifungal stewardship^26^.

Despite these advantages, translational challenges require attention. First, at near-LOD concentrations, variable T1/T2 line intensities (Fig. 4) may introduce visual interpretation bias; smartphone-based colorimetric algorithms could enable quantitative analysis^27^. Second, current probes may not cover rare ITS2 variants (e.g., *C. krusei* haplotype B), warranting expanded clinical validation for genotypic inclusivity. Finally, matrix tolerance for whole-blood samples—key for bloodstream infections—remains unassessed. To address these, future work will focus on: ① Lyophilized bead-based reagents for ambient stability; ② Expanding to 5–7-plex detection covering pan-resistant species including *C. auris*; ③ Integration with microfluidics for automated sample processing. We anticipate that with the growing accessibility of portable nucleic acid extractors and CRISPR signal amplification^28^ such RPA-LFS systems may redefine fungal diagnostics and ultimately be included in WHO’s POCT recommendations for drug-resistant pathogens.

## Conclusion

This study established a dual-labeled probe-based RPA-LFS system for simultaneous rapid identification of *C. glabrata* and *C. krusei*. Through optimized ITS2-targeted primers/probes and integrated dual test lines, the system delivers “sample-to-result” output within 30 min. Validation demonstrated: detection limits down to 10² copies/mL, 100% inclusivity across 15 target strains, strict discrimination from 14 non-target pathogens (100% specificity), and perfect concordance (100%) with qPCR in 328 clinical samples. Requiring no complex instrumentation and enabling visual readout, this system provides an efficient tool for point-of-care management of drug-resistant Candida infections and rational antifungal therapy, particularly in primary healthcare settings.

## Data Availability

The original contributions presented in the study are included in the article. Further inquiries can be directed to the corresponding authors.
